# Probing the Effector and Suppressive Functions of Human T Cell Subsets Using Antigen-Specific Engineered T Cell Receptors

**DOI:** 10.1371/journal.pone.0056302

**Published:** 2013-02-20

**Authors:** Qi Wan, Lina Kozhaya, Keren Imberg, Frances Mercer, Shi Zhong, Michelle Krogsgaard, Derya Unutmaz

**Affiliations:** 1 Department of Microbiology, New York University School of Medicine, New York, New York, United States of America; 2 Department of Pathology, New York University School of Medicine, New York, New York, United States of America; 3 Department of Medicine, New York University School of Medicine, New York, New York, United States of America; 4 New York University Cancer Institute, New York University School of Medicine, New York, New York, United States of America; University of California, San Francisco, United States of America

## Abstract

Activation of T cells through the engagement of the T cell receptors (TCRs) with specific peptide-MHC complexes on antigen presenting cells (APCs) is the major determinant for their proliferation, differentiation and display of effector functions. To assess the role of quantity and quality of peptide-MHC presentation in eliciting T cell activation and suppression functions, we genetically engineered human T cells with two TCRs that recognize HLA-A*0201-restricted peptides derived from either HIV or melanoma antigens. The engineered-TCRs are highly functional in both CD8^+^ and CD4^+^ T cells as assessed by the upregulation of activation markers, induction of cytokine secretion and cytotoxicity. We further demonstrated that engineered-TCRs can also be expressed on naïve human T cells, which are stimulated through APCs presenting specific peptides to induce T cell proliferation and acquire effector functions. Furthermore, regulatory T cells (Tregs) ectopically expressing the engineered-TCRs are activated in an antigen-specific fashion and suppress T cell proliferation. In this system, the inhibitory activity of peptide-stimulated Tregs require the presence of dendritic cells (DCs) in the culture, either as presenters or as bystander cells, pointing to a critical role for DCs in suppression by Tregs. In conclusion, the engineered-TCR system reported here advances our ability to understand the differentiation pathways of naïve T cells into antigen-specific effector cells and the role of antigen-specific signaling in Treg-mediated immune suppression.

## Introduction

Human T cells engineered to express T cell receptors (TCRs) specific for antigens from tumors or infectious organisms have recently been developed as an effective adoptive immunotherapy [1]–[3]. Infusion of genetically reprogrammed T cells recognizing tumor antigens into patients has had reasonable success in augmenting tumor-specific immunity [1], [2] and can confer some ability for tumor regression [2], [3]. More recently, this engineering approach was applied to generate CD8^+^ T cell responses to HIV antigens as a potential therapeutic approach to eliminate virus-infected cells in patients [4]–[6]. However, TCR-transduced CD8^+^ T cells derived from effector T cells appear to be short-lived *in vivo* [7]. Thus, it may be preferable to maintain the naïve phenotype of TCR-engineered T cells *in vitro*. Indeed, it was shown that antigen-specific effector T cells derived from human naïve CD8^+^ T cells, compared to memory T cells, resist terminal differentiation and maintain high proliferative capacity [8]. Therefore, antigen-specific TCR-engineered naïve T (T_N_) cells with enhanced proliferative capacity, differentiation and better survival are needed to improve both *in vivo* cell therapies and vaccine formulations.

The TCR-engineering strategy can also be applied to understand how quantitative and qualitative signals from the TCR impact the suppressive function of human regulatory T cells (Tregs), which are required for controlling unwanted T cell responses to self or foreign antigens. Recently it was shown that Tregs could also be genetically modified using engineered-TCRs [9]–[11]. In mouse models, these TCR-engineered Tregs effectively blocked antigen-specific effector T cell activity and promoted transplantation tolerance [10]. However, the activation parameters and the requirements of antigen presenting cells (APCs) that control Treg suppressive function remain unclear. Therefore, the engineered-TCR approach can be a powerful tool to better understand the biology of human Tregs with the goal to optimize the utility of Tregs in adoptive therapies.

In this study, we engineered two different mouse-human hybrid TCRs (referred to as engineered-TCRs) specific for melanoma (gp100) and HIV (SL9) antigens [6], [12]. Expression of these engineered-TCRs in primary human CD4^+^ and CD8^+^ T cells revealed that gp100-TCR-transduced cells have a lower threshold, compared to SL9-TCR-expressing cells, for activation and induction of effector functions. We then introduced the engineered-TCRs into T_N_ cells cultured in IL-7 and showed that naïve CD8^+^ and CD4^+^ T cells cultured in IL-7 could be transduced to express the engineered-TCRs and stimulated by specific peptide-presenting APCs to proliferate and display antigen-specific effector functions. In addition, we determined that antigen-specific TCR transduced Tregs could suppress antigen-specific T cell activation, regardless of whether they recognize the same antigens or different ones. Significantly, Tregs exhibit a strong requirement for the presence of dendritic cells (DCs) in culture to display their inhibitory activity. Thus, this engineered-TCR strategy can be used to determine the antigen-specific activation and differentiation requirements of human effector and regulatory T cell subsets and may be applied to infectious diseases such as HIV infection, for development of vaccines and adoptive anti-tumor therapeutic approaches.

## Materials and Methods

### Ethics Statement

Discarded buffy coats from healthy individuals were obtained anonymously from New York Blood Center, New York, NY. All donor samples were non-identifiable and did not involve any donor-specific information for data analysis and therefore consent forms were not required. All human material was obtained and processed according to guidelines and approval of NYU School of Medicine Institutional Human Subjects Board.

### Human T Cell Purification and Activation

Peripheral Blood Mononuclear Cells (PBMCs) from healthy individuals were prepared using Ficoll-paque plus (GE Amersham, Uppsala, Sweden). CD4^+^ T cells were isolated using Dynal CD4 Positive Isolation Kit (Invitrogen, Carlsbad, CA) directly from purified PBMCs and were >99% pure. Purified CD4^+^ T cells were sorted by FACS (ARIA cell sorter, BD Biosciences) into CD45RO^−^CD25^−^ T_N_, CD45RO^−^CD25^+^ naïve regulatory T (TNregs) cells. T_N_ and TNregs were activated by anti-CD3 and anti-CD28 (aCD3/aCD28) coated beads (1∶4 beads:cells ratio) and cultured in RPMI 1640 medium complemented with 20 ng/ml IL-2 (R&D Systems, Minneapolis, MN), 10% FBS (Fetal Bovine Serum) (Atlanta biologicals, Lawrenceville, GA). TNregs differentiate into mature Tregs after *in vitro* expansion and show potent suppression of T cell activation [13], [14]. Alternatively, T_N_ cells were maintained in 20 ng/ml IL-7 (R&D Systems) medium for duration of indicated days. Monocyte-derived DCs from healthy donors were generated from CD14^+^ cells as previously described [15]. The following peptides were used to activate TCR-engineered T cells: HIV Gag epitope, SL9 (also called p17, amino acid 77–85, SLYNTVATL) (Genescript, Piscataway, NJ); Melanoma antigen, glycoprotein 100∶210M (gp100, amino acid 209–217, with a methionine substitution at position 210, IMDQVPFSV). Melanocyte differentiation antigen recognized by T cells (MART-1) was used as a control peptide, Mart-1∶27L (amino acid 26–35, with a leucine substitution at position 27, ELAGIGILTV) (Bio-Synthesis Inc, Lewisville, TX). These HLA-A*0201-restricted peptides were presented to T cells using either T2 cells, a human lymphoblastoid cell line deficient in genes for transporters associated with antigen presentation (TAP1/TAP2) but expressing HLA-A*0201 molecules [7], [16], [17], or lipopolysaccharide (LPS, 10 ng/ml)-stimulated HLA-A*0201^+^ monocyte-derived DCs.

### Cloning of the Peptide-specific Mouse-human TCR

A single TCR sequence linked by a picornavirus-like 2A “self-cleaving” peptide (1803 TCRα-2A-TCRβ) specific against HIV SL9 peptide was a gift from Dr. Harris Goldstein’s lab [6]. This construct was used as the template for PCR-cloning of human SL9-TCR variable regions, similar to previously described methods [12] and cloned into an HIV derived lentiviral vector (HDV). As shown in Figure S1, 6 primers were designed and 3-step-PCRs were performed to create final TCR-lentiviral vector against HIV SL9-peptide from two templates mentioned above. Engineered-TCR against Melanoma gp100 epitope was cloned from a human-TCR specific for melanoma gp100 peptide (a gift from Dr. Steven A. Rosenberg) using similar strategy (data not shown). The primers for SL9-TCR amplification are:

primer#1:

5′ATAGCGGCCGCGCCACCATGATGAAATCCTTGAGAGT3′;

primer#2:

5′CAGGTTCTGGGTTCTGGATGTTTGAAATCACAGAAAGTCTTGTG3′;

primer#3:

5′GTCTCCTGCTTGCTTTAACAGAGAGAAGTTCGTGGCTCCGGAGCCACTGGACCACAGCCTCAGCGTCATGAG3′;

primer#4∶5′TTCTCTCTGTTAAAGCAAGCAGGAGACGTGGAAGAAAACCCCGGTCCATGGGCTCCAGGCTGCTCTGTTGGGTG3′;

primer#5∶5′CACCAGGCTCACGGTCACAGAGGATCTGAGAAATGTGACTCCAC3′;

primer#6:

5′ATACAATTGTCAGGAATTTTTTTTCTTGACCATG3′.

### Lentivirus Production and Transduction

The lentiviruses pseudotyped with VSVG envelope were generated as previously described [18]. All lentiviruses express GFP and RFP as the marker in place of the *nef* gene [18]–[20]. Viral titers were measured using same method described [18], [20] and ranged from 1–30×10^6^ IFU/ml. Activated T_N_ cells and TNregs were transduced with engineered-TCRs at day 1 post aCD3/aCD28 beads activation and were maintained in IL-2 containing medium for 14 days, and subsequently sorted for GFP^+^ or RFP^+^ T cells. IL-7 cultured resting T_N_ cells were transduced with engineered-TCRs at day 7 post IL-7 and were kept in the same culture for another 7 days before stimulation with specific peptides. In HIV-target co-culture experiments, activated CD4^+^ T cells were transduced with VSVG pseudotyped HIV virus encoding RFP 1 day after aCD3/aCD28 beads activation.

### Staining and FACS Analysis

Cell surface staining was performed as previously described [20]. Briefly, cells were stained with relevant antibodies on ice for 30 min in PBS buffer containing 2% FBS and 0.1% sodium azide in dark. Cells were washed twice then analyzed by flow cytometry using BD LSR-II (BD Biosciences, San Jose, CA). Live cells were gated based on Fixable Viability Dye eFluor450 (eBioscience, San Diego, CA). For intracellular staining, after surface staining, cells were fixed and permeabilized by commercially available Foxp3 intracellular staining kit (eBioscience, San Diego, CA) as per manufacturer’s protocol. After permeabilization and fixation, cells were washed twice with the permeabilization buffer (eBioscience, San Diego, CA) and incubated with corresponding antibodies at 4°C for 30 min. Cells were washed twice again with the permeabilization buffer before FACS analysis. Antibodies used for surface and intracellular staining included: CD45RO-Pacific Blue, CD25-APC, CCR7-FITC, GARP-APC, Foxp3-A488, Helios-PE (all from Biolegend, San Diego, CA); anti-mouse TCR-constant β-APC or PE (eBioscience); MHC Dextramer A0201/SLYNTVATL-APC and MHC Dextramer A0201/IMDQVPFSV-APC were obtained from Immudex, Copenhagen, Denmark.

### Cell Proliferation and Cytokine Assays

Cells were labeled with CellTrace Violet or CFSE (Invitrogen) following manufacturer’s instruction and activated with different concentrations of peptides presented by DCs or T2 cells. Supernatant from the culture were collected 16–40 hr later. Cytokine production was measured using Cytometric Bead Array (CBA; BD Biosciences). Proliferation of the cells was assessed using FACS analysis of dilution of CellTrace Violet or CFSE labeling on day 4-, day 5-, and day 6- post activation of the T cells.

### *In vitro* Suppression Assay

Suppression assays were set up as previously described [21], [22]. Briefly, Resting CD4^+^ T_N_ cells were sorted as described above and were cultured in IL-7 for 7 days followed by gp100-TCR or SL9-TCR transduction. After 2 weeks of culture in IL-7, T_N_ cells were labeled with CellTrace Violet and were used as targets. Labeled resting T_N_ cells and non-labeled gp100-TCR-transduced Tregs were mixed at 1∶1 T_N_:Tregs ratio. The cells were stimulated by different peptides presented by T2 cells and/or DCs (at 1∶1 ratio of Treg:T2 or Treg:DC), The peptide concentrations for target cells ranged between 0–1000 nM and the concentration of gp100 for Tregs_TCR-gp100_ ranged from 0–10,000 nM in final cell culture. Cells were harvested and analyzed by BD LSR II on day 4- or day 5- post activation.

### Data Analysis

FACS data was analyzed using FlowJo (Tree Star, Ashland, OR) and statistical analysis was performed using Graphpad prism software (Graphpad Inc., La Jolla, CA).

## Results

### Expression of Engineered Antigen-specific TCR in Human T Cell Subsets

**Figure 1:**
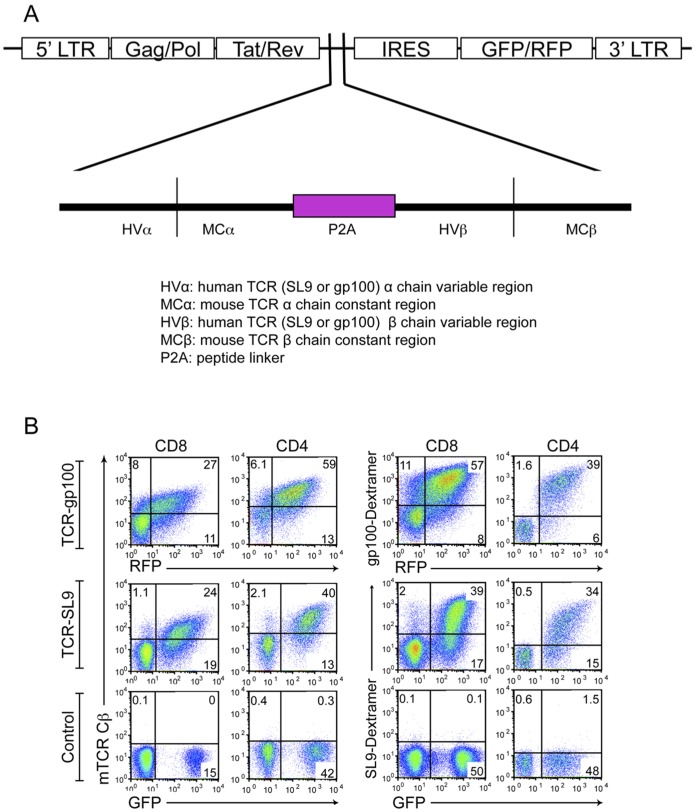
Expression of mouse-human hybrid gp100-TCR or SL9-TCR on primary human T cell subsets. (A) Schematic diagram of the generation of TCR-SL9 or TCR-gp100 lentiviral constructs. Mouse-human hybrid TCRs were generated by linking human TCR variable regions for SL9 or gp100 to mouse TCR constant region in a single sequence linked by a Picornavirus-Like 2A “Self-Cleaving” Peptide (P2A). **(B)** Validation of the expression of TCRs by T cells. Different primary human T cell subsets were activated and transduced with distinct TCRs, SL9-TCR (GFP) or gp100-TCR (RFP). T cells were stained with either mouse TCR constant region β chain antibody (left) or Dextramers (right). The data are representative from three different experiments from multiple donors.

T cells engineered with mouse-human TCRs were shown to have higher cell surface expression and increased TCR complex stability, compared to T cells engineered with human TCRs (hTCRs) alone, which results in an enhanced anti-tumor activity [12]. Therefore, we used this approach to generate engineered-TCRs specific for either HIV SL9 or Melanoma gp100 peptides in lentiviral vectors **(** **Figure 1A** **and Figure S1**). We then expressed both engineered-TCRs (hereafter referred to as “SL9-TCR” or “gp100-TCR”) in activated human CD8^+^ and CD4^+^ T cells and confirmed the expression using either antibodies against mouse TCR constant region β (mTCR Cβ) or peptide pulsed dextramers **(** **Figure 1B** **)**.

**Figure 2:**
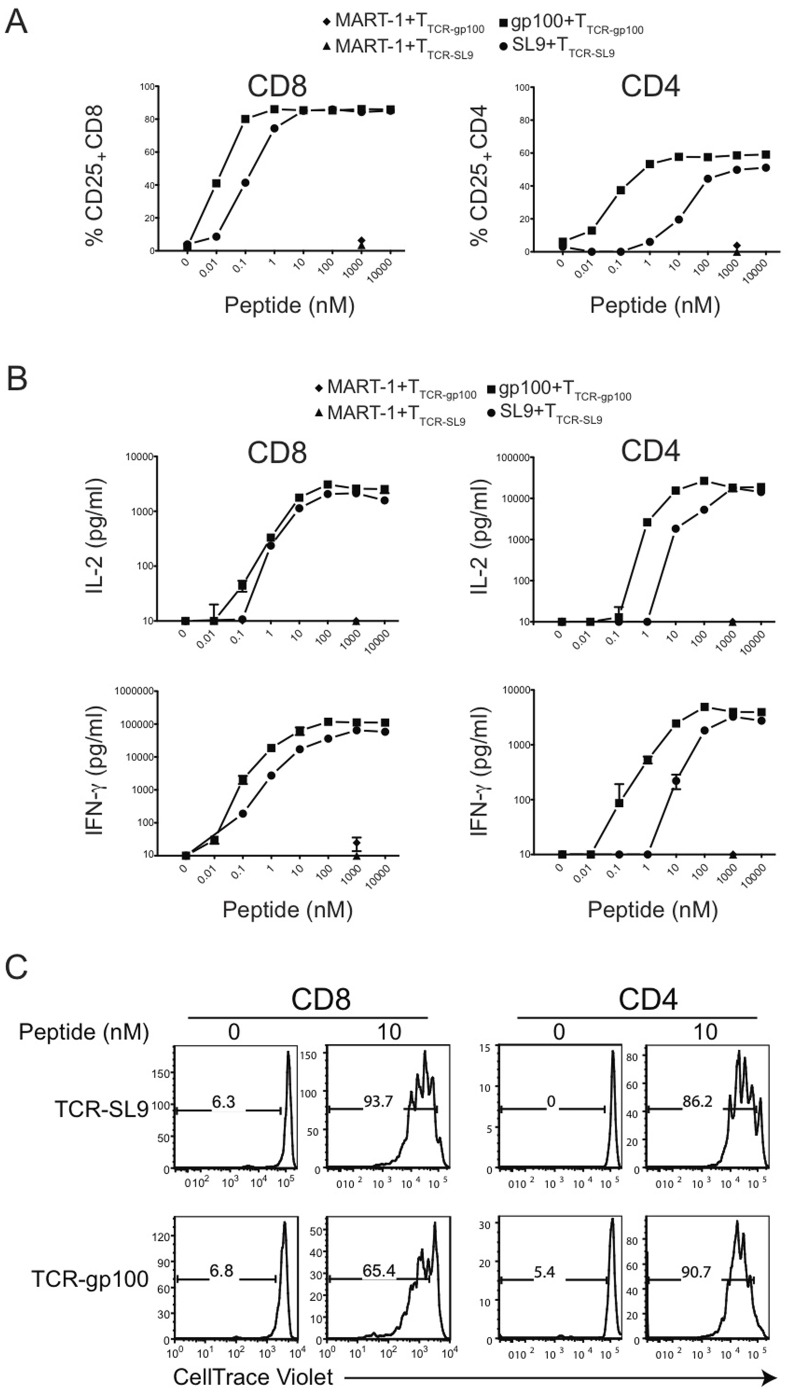
Peptide-dependent activation of human T cell subsets engineered with specific TCRs. (**A**) Upregulation of CD25 expression from T cells expressing gp100-TCR or SL9-TCR in a dose-dependent manner. CD8^+^ or CD4^+^ T cells were activated and transduced with distinct TCRs. Sorted pure T_TCR-gp100_ or T_TCR-SL9_ cells were cultured with either gp100 or SL9 presented through T2 cells. The frequency of CD25 expressing cells was determined by FACS analysis. The data are representative from three different experiments from multiple donors. (**B**) Concentration-dependent induction of IL-2 and IFN-γ secretion from gp100-TCR or SL9-TCR-transduced T cells. Cytokines were measured in the supernatant taken from (A) using CBA assay. The data represent the mean±SD from three different donors. (C) Antigen-specific proliferation of gp100- or SL9- TCR engineered T cells as in (A) was monitored. The data are representative from three different experiments from multiple donors.

The T2 cell line, derived from B lymphoblastoid cell line (B-LCL) 721.174 fused with T-LCL, has a large deletion in the MHC class-II region. The deletion includes genes for TAP1/TAP2 [7], [16], [17], [23]. As such, most MHC class-I molecules on T2 cells are devoid of peptides and they can be stabilized by engagement with exogenous peptides [24], [25]. Stimulation of both CD4^+^ and CD8^+^ T cells expressing either gp100-TCR (referred to as CD4_TCR-gp100_ or CD8_TCR-gp100_) or SL9-TCR (referred to as CD4_TCR-SL9_ or CD8_TCR-SL9_) through corresponding peptide pulsed HLA-A*0201-expressing T2 cells [26] upregulated CD25 expression in a peptide concentration-dependent manner **(** **Figure 2A** **)_._** We observed that 50% of gp100-TCR-expressing T cells upregulated CD25 at 10 or 100 times lower peptide concentration in comparison to SL9-TCR-expressing T cells **(** **Figure 2A** **)**, suggesting a relatively higher affinity for gp100-TCR to peptide-MHC complexes.

While the TCRs used in this study were MHC class-I restricted, CD4^+^ were also efficiently activated **(** **Figure 2A** **)** and secreted IL-2 and IFN-γ in response to corresponding peptide stimulation in a dose-dependent manner **(** **Figure 2B** **)**, similar to engineered-TCR transduced CD8^+^ T cells **(** **Figure 2B** **)** and consistent with previous reports [27], [28]. However, upon stimulation with T2 cell-presented peptides, CD4^+^ T cells produced higher amount of IL-2 compared to CD8^+^ T cells, whereas CD8^+^ T cells secreted relatively more IFN**-γ (** **Figure 2B** **)**. Stimulation of both T cell subsets by peptide-pulsed T2 cells also resulted in their proliferation **(** **Figure 2C** **)**. T cells stimulated with a non-recognized control peptide (MART-1) neither upregulated CD25 nor secreted any cytokines **(** **Figure 2A and 2B** **)**. Moreover, compared to human TCR that lack mouse constant regions, the mouse-human hybrid TCR used in this study allowed T cells to secrete higher levels of cytokines at similar concentration of peptide stimulation **(Figure S2)**.

### Cytotoxic Effector Function of TCR-engineered T Cells

**Figure 3:**
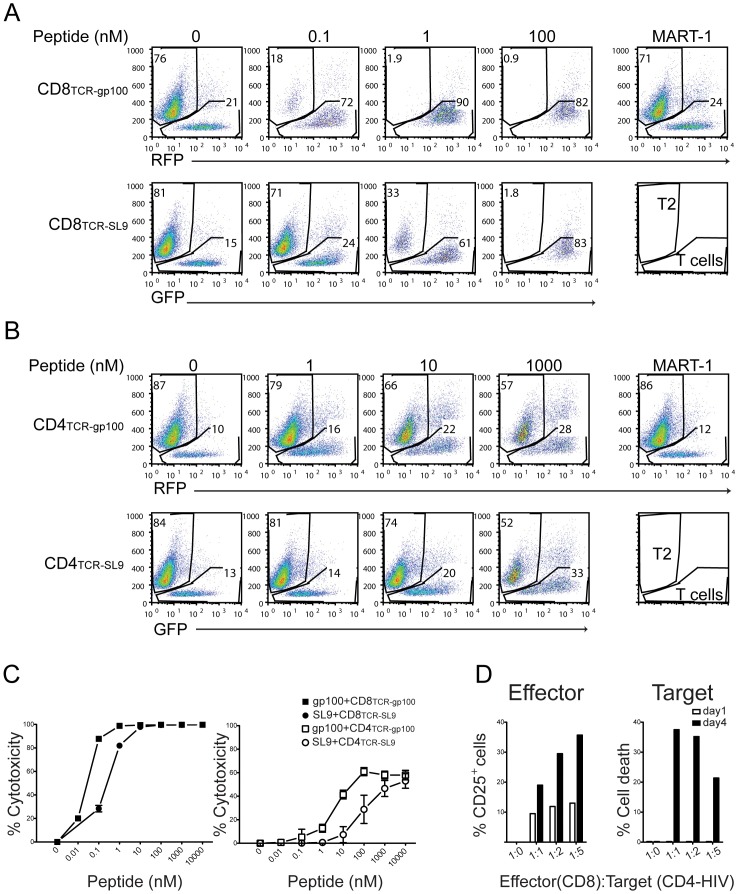
Antigen-specific cytotoxicity of T cells expressing engineered-TCRs. CD8^+^ and CD4^+^ T cells expressing gp100-TCR or SL9-TCR are cytotoxic in a peptide concentration-dependent fashion. Activated CD8^+^ (**A**) or CD4^+^ (**B**) T cells ectopically expressing gp100-TCR or SL9-TCR were sorted based on GFP or RFP expression. Sorted cells were cultured with T2 cells plus different peptides at the indicated concentrations. The portion of viable T2 cells was analyzed by FACS after activation, as shown on the Y-axis, whereas the percentage of T cells is shown on the X-axis (**A, B**). MART-1 (1000 nM) was used as a control. The data are representative from three different experiments from multiple donors. (**C**) The percent killing of T2 cells (% Cytotoxicity) by CD8^+^ T cells. We calculated % Cytotoxicity by comparing percentage of T2 cell death from peptide-cultured group to those from no peptide control group. The data represent the mean±SD from three different donors. (**D**) CD8_TCR-SL9_ effector cells kill HIV-infected CD4^+^ T cells. HLA-A*0201^+^ CD4^+^ T cells were infected with VSVG.HIV, and were co-cultured with CD8_TCR-SL9_ at different CD8^+^: CD4^+^ cell ratios as indicated. The upregulation of CD25 on CD8^+^ T cells (Effector) as well as the % cell death of CD4^+^ T cells (Target), normalized to a CD4-HIV T cell-only group, was determined by FACS analysis. The data are representative from three different experiments from multiple donors.

We next determined the cytotoxic functions of the engineered-TCR-transduced T cells. In this experiment, T2 cells presenting respective peptides were used as target cells for cytotoxicity. We found that T2 cells were killed either by CD8_TCR-gp100_ or CD8_TCR-SL9_ in a dose-dependent manner with their respective peptides **(** **Figure 3A and 3C** **)**. Similar to CD25 expression and cytokine production, compared to SL9 peptide, 10–100 times less gp100 peptide concentration was sufficient to induce cytotoxic function **(** **Figure 3A and 3C** **)**. This cytotoxicity was antigen-specific and was not observed with control MART-1 peptides **(** **Figure 3A and 3C** **)**. The efficacy of cytotoxicity by CD8^+^ T cells towards T2 cells was most optimal at the highest 1∶1 ratio tested (**Figure S3**).

It has been shown that, similar to CD8^+^ cells, CD4^+^ cells harboring human MHC class I- restricted specific TCRs exhibit anti-tumor activity *in vivo* and *in vitro* and are suggested to be as efficacious as CD8^+^ T cells [27], [28]. Therefore, we determined the cytotoxic functions of gp100- or SL9-TCR-engineered CD4^+^ effector T cell subsets. We found that TCR-engineered CD4^+^ T cells were much less cytotoxic towards peptide-pulsed T2 cells and required higher levels of peptide (∼100–1000 times), in comparison to CD8^+^ T cells **(** **Figure 3B and 3C** **)**. These results suggest that, although CD8 molecules expressed on the T cell surface better facilitate the engagement of TCR-peptide-MHC Class-I clusters and enhance the functional capacity of CD8^+^ T cells, CD4^+^ T cells expressing specific TCRs can also elicit low-level cytotoxic function but require much higher peptide concentrations relative to CD8^+^ T cells.

The artificial human TCR-engineered CD8^+^ T cells specifically targeting the HIV Gag epitope, SL9, were shown to potently inhibit HIV infectivity and replication *ex vivo* [4], [6], [29]. We therefore asked to what extent the CD8_TCR-SL9_ could kill primary CD4^+^ T cells infected with HIV. To directly address this question, we infected HLA-A*0201^+^ CD4^+^ T cells with VSVG-pseudotyped HIV (VSVG.HIV) virus that expresses Gag protein containing SL9 peptide, and evaluated whether CD8_TCR-SL9_ could kill CD4^+^ T cells endogenously expressing HIV antigens (CD4-HIV). We found that CD8_TCR-SL9_ co-cultured with CD4-HIV were activated as indicated by the upregulation of CD25 at 1 or 4 days post co-culture proportionate to higher ratios of CD4-HIV T cells **(** **Figure 3D** **)**. After 4 days of co-culture at 1∶1 ratio of effectors and target cells, about 40% of CD4-HIV cells were killed by CD8_TCR-SL9_ **(** **Figure 3D** **)**.

### Generation of Antigen-specific Naïve T Cells

**Figure 4:**
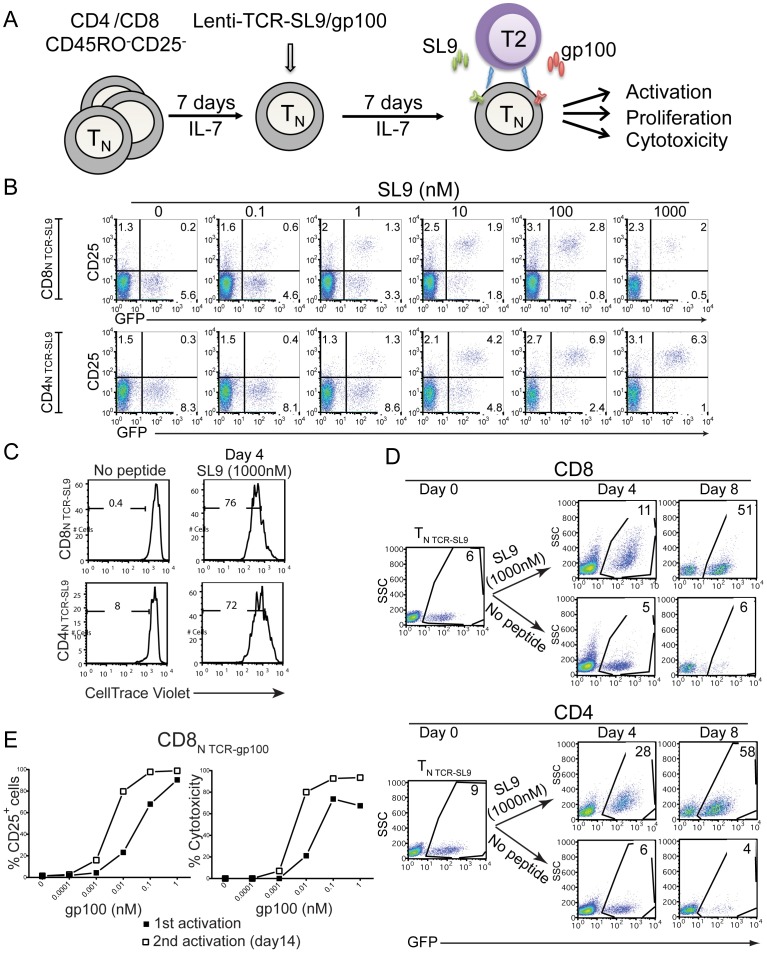
Antigen specific activation and proliferation of naïve human T cells transduced with engineered-TCRs. (**A**) Resting CD4^+^ and CD8^+^ CD45RO^−^CD25^−^ T (T_N_) cells were cultured in IL-7-containing medium for 7 days followed by ectopic expression of SL9- or gp100-TCR. 7 days later, cells were then activated with either SL9 or gp100 presented by T2 cells. (**B**) Upregulation of CD25 from T_N_ cells expressing SL9-TCR upon activation. Cells collected as indicated in (A) were subject to CD25 staining followed by FACS analysis. The data are representative from three different experiments from multiple donors. (**C, D**) CD8^+^ and CD4^+^ T_N_ cells expressing SL9-TCR proliferate and expand via activation through SL9 peptide presented by T2 cells. SL9-TCR-transduced cells as indicated in (A) were labeled with CellTrace Violet and the proliferation was monitored at day 4 post activation and the expansion of T cells was determined at day 4 and day 8 post activation. (**E**) Induction of CD25 and cytotoxicity from gp100-TCR-transduced CD8^+^ T_N_ cells at 1st TCR-stimulation and during reactivation. gp100-TCR-overexpressing CD8^+^ T_N_ cells were co-cultured with T2 cells in the presence of gp100. The frequency of CD25^+^ cells and the cytotoxicity were determined 1 day after 1st activation. Activated cells were kept in culture for an extra 2 weeks and were then restimulated again with gp100 presenting T2 cells (2^nd^ activation). CD25 levels and cytotoxicity were determined 1 day after reactivation. The data are representative from three different experiments from multiple donors.

We have previously shown that common gamma-chain (γc)-utilizing cytokines, such as IL-7, IL-15 and IL-2 can promote T cell survival in the absence of TCR signaling without inducing significant cell division [30] and that γc-cytokine-culture renders resting naïve T cells susceptible to HIV infection or transduction [18]. To investigate the possibility that resting naïve T cells can be directed into a unique, antigen-specific effector cell population using engineered-TCRs, we rendered T_N_ cells susceptible to TCR-transduction by a 7-day culture in IL-7-containing medium **(** **Figure 4A** **)**. We then ectopically expressed engineered-TCRs and determined their effector functions as shown in the schematic **(** **Figure 4A** **)**. Naïve CD4^+^ T cells (CD4_N TCR-SL9_/CD4_N TCR-gp100_) (data not shown) and CD8^+^ T cells (CD8_N TCR-SL9_/CD8_N TCR-gp100_) **(Figure S4)** expressed the engineered-TCRs and maintained their naïve phenotype. Both CD4_N TCR-SL9_ and CD8_N TCR-SL9_ cells upregulated CD25 in response to SL9 presented by T2 cells **(** **Figure 4B** **)**. In addition, stimulation of SL9-TCR engineered T_N_ cells with the optimal level of peptide (1 µM SL9) induced efficient proliferation **(** **Figure 4C** **)**. Remarkably, both CD4_N TCR-SL9_ and CD8_N TCR-SL9_ expanded about 5–10 fold over a period of 8 days in IL-2 containing medium **(** **Figure 4D** **)**. These findings indicate that the engineered-TCR approach can be used to generate antigen-specific naïve human T cells to study their proliferative capacity and differentiation *in vitro*.

We next addressed the ability of CD8^+^ T_N_ cells, transduced with engineered-TCRs, to display cytotoxic function upon specific-peptide stimulation. In this experiment, we used CD8_N TCR-gp100_ because they were more efficient in mediating cytotoxicity compared to SL9-TCR-engineered cells **(** **Figure 3** **)**. Stimulation of CD8_N TCR-gp100_ with gp100 presented by T2 cells resulted in a dose-dependent increase in CD25 expression (1^st^ activation) **(** **Figure 4E, left panel** **)**. When these T_N_ cells were expanded for 2 weeks and then restimulated with the same peptide presented by T2 cells, they required about 10 fold lower peptide dose for comparable expression of CD25 (2^nd^ activation) **(** **Figure 4E, left panel** **)**. It should be emphasized that gp100-TCR engineered CD8^+^ T cells displayed significant cytotoxicity even during the first stimulation **(** **Figure 4E, right panel** **)**. Similarly, CD8_N TCR-gp100_ required 10 times less peptide dose for their cytotoxicity on second activation compared to the first one **(** **Figure 4E, right panel** **)**. Taken together, these findings suggest that T_N_ cells can be directed into antigen-specific cytotoxic CD8^+^ T_N_ cells through the engineered-TCR approach and peptide-specific stimulation.

### TCR-engineered Tregs are Suppressive *in vitro*

**Figure 5:**
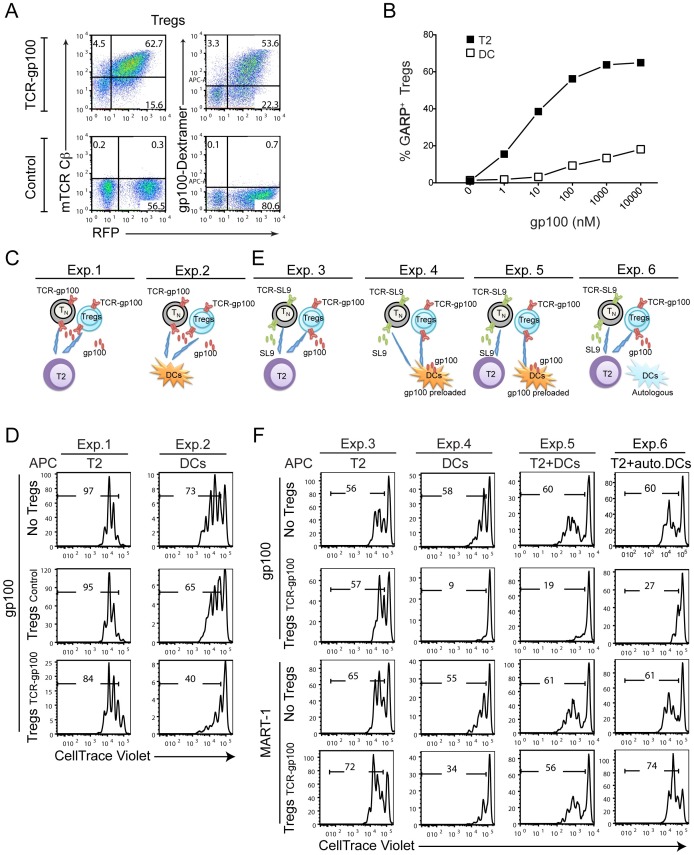
Suppressive function of TCR-engineered Tregs. (**A**) Validation of the expression of gp100-TCR-engineered Tregs. gp100-TCR overexpressing Tregs were stained with mTCR constant β chain antibody or gp100-dextramer and analyzed by FACS. (**B**) Tregs_TCR-gp100_ upregulate GARP upon peptide activation. Tregs expressing gp100-TCR were stained with a GARP antibody 2 days after gp100 presentation by T2 cells or DCs. A dose-dependent upregulation of GARP on Tregs_TCR-gp100_ is shown. The data are representative from three different experiments from multiple donors. (**C–F**) Tregs_TCR-gp100_ suppress the proliferation of T_N TCR-gp100_ and T_N TCR-SL9_ cells *in vitro*. At 2 weeks post activation, expanded gp100-TCR-transduced Tregs were sorted by FACS, using RFP expression as a marker. IL-7 cultured, gp100- or SL9-TCR-engineered, and CellTrace violet dye-labeled T_N_ cells were used as target cells. Suppression assay was set up at Tregs:target 1∶1 ratio, either using same peptide (**C, D**) or different peptides (**E, F**). The proliferation of target cells was determined at day 5 post activation. (**C, D**) Tregs_TCR-gp100_ were mixed with labeled T_N TCR-gp100_ in the presence of 10 nM gp100 presented by either T2 cells **(Exp.1)** or DCs **(Exp.2)**. (**E,F**) Tregs_TCR-gp100_ were mixed with labeled T_N TCR-SL9_ when gp100 and SL9 were presented by either T2 cells **(Exp. 3)** or HLA-A*0201^+^ DCs **(Exp. 4)**. In **Exp.4**, DCs were preloaded with gp100 (10,000 nM) 1 day in advance and were washed three times before co-culturing with Tregs and T_N_ cells. In **Exp.5**, Tregs_TCR-gp100_ were activated with gp100 preloaded DCs and T_N TCR-SL9_ were activated with SL9 presented by T2 cells. In **Exp.6**, suppression assay was set up as in **Exp.3** except that autologous DCs of Tregs (HLA-A*0201^−^) were added to the co-culture. The data are representative from three different experiments from multiple donors.

Antigen-specific studies in human Tregs are challenging due to the difficulty in expanding clonal Tregs *in vitro* [31]. Recently it was reported that engineered-TCRs could redirect polyclonal Tregs in response to a specific antigen *in vivo* [9], [10]. To study the activation and suppression requirements of human Tregs, we used a similar strategy to express an engineered-TCR (gp100-TCR) in human Tregs (Tregs_TCR-gp100_) **(** **Figure 5A** **)**. Tregs_TCR-gp100_ were >90% pure, and expressed both FOXP3 and HELIOS **(Figure S5A)** transcription factors [32]–[34]. In order to determine the response of gp100-TCR-Tregs to peptide-specific activation, we monitored the induction of the Treg specific molecule, GARP, which is upregulated upon TCR-triggering specifically on Treg cells [35]. GARP was upregulated on Tregs_TCR-gp100_ upon peptide stimulation, in a dose-dependent manner **(Figure S5A)**. T2 cells were more efficient than DCs in upregulating GARP expression on Tregs_TCR-gp100_ **(** **Figure 5B** **)**, possibly due to higher levels of empty MHC Class-I molecules on T2 cells. T2 cells pulsed with gp100 peptide also induced proliferation of Tregs_TCR-gp100_ similar to T_TCR-gp100_ cells **(Figure S5B)**. However, proliferation of Tregs_TCR-gp100_ was less robust when stimulated with DCs pulsed with gp-100 peptide, compared to T_TCR-gp100_ cells **(Figure S5B)**. In both of these stimulation conditions Tregs secreted much less IL-2 compared to conventional T cells **(Figure S5C)**.

We next set up a series of experiments to investigate several key questions on the nature of antigen-specific suppression mediated by Tregs **(** **Figure 5C–F** **)**. In the first set of experiments, we asked whether Tregs_TCR-gp100_ could suppress the proliferation of T_N TCR-gp100_ when same antigen (gp100) was presented either by T2 cells **(** **Figure 5C** **, Exp.1)** or by DCs **(** **Figure 5C** **, Exp.2)**. We found that Tregs effectively suppressed target T cell proliferation, when DCs were used as APCs **(** **Figure 5D** **, Exp. 2)** but not when T2 cells were used as APCs for both targets and Tregs **(** **Figure 5D** **, Exp. 1)**. This finding suggests that TCR-engineered Tregs are capable of suppressing the proliferation of target cells when these cells are both activated by the same antigen only when DCs are APCs.

We then asked whether Tregs were suppressive when different peptides (gp100 for Tregs and SL9 for target T cells) were presented by the same T2 cells **(** **Figure 5E** **, Exp.3)** or through DCs **(** **Figure 5E** **, Exp.4)**. In experimental setup 3 (Exp.3), Tregs_TCR-gp100_ activated by T2 cells presenting gp100 peptide did not suppress the proliferation of T_N TCR-SL9_ cells, which were activated by the SL9 peptide **(** **Figure 5F** **)**. In contrast, when DCs were used as APCs in a similar experiment **(** **Figure 5F** **, Exp. 4)**, gp100-peptide stimulated Tregs_TCR-gp100_ potently suppressed target T_N TCR-SL9_ cell proliferation, induced by a different antigen (SL9 peptide) **(** **Figure 5F** **, Exp.4)**. Low-level suppression of target T cell proliferation was also observed in non-stimulating control MART-1 peptide condition **(** **Figure 5F** **, Exp.4)**, likely due to the activation of Tregs_TCR-gp100_ by allogeneic DCs used in this experiment **(** **Figure 5F** **, Exp.4)**. Together, these different experimental setups suggest that presentation of the antigen to Tregs by DCs amplify their suppressive capability.

To determine whether Tregs_TCR-gp100_ could display suppressive function when different peptides were presented by distinct APCs, gp100-preloaded DCs were used to stimulate Tregs_TCR-gp100_ and SL9-pulsed T2 cells to activate target T cell proliferation **(** **Figure 5E** **, Exp.5)**. In this experiment, Tregs_TCR-gp100_ also suppressed the proliferation of T_N-TCR-SL9_ **(** **Figure 5F** **, Exp.5)**. In the final set of experiment, we evaluated whether the DCs are required in this system to activate Tregs, or whether they could provide signals in a bystander fashion. Accordingly, we setup an approach similar to experiment 3 (Exp.3) where both target T cells and Tregs were stimulated by two separate peptides presented by T2 cells, whereas autologous HLA-A*0201^−^ DCs, which are derived from the same donor as Tregs and thus cannot present the specific peptides, were added as bystander cells **(** **Figure 5E** **, Exp.6)**. Remarkably, when Tregs were stimulated by peptide-presenting T2 cells, the presence of the bystander autologous DCs was sufficient to render Tregs_TCR-gp100_ suppressive **(** **Figure 5F** **, Exp.6)**. Collectively, these experiments reveal that the suppressive capacity of antigen-specific TCR-engineered Tregs is highly dependent on the presence of DCs, either as presenters of antigen or as bystander cells.

## Discussion

In this study, we demonstrate the utility of engineered-TCRs with known specificities to study the activation requirements of naïve human T cells and antigen-specific suppression mechanisms of human Tregs. Our findings indicate that the modified genetic TCR gene transfer techniques established in our study can be a useful tool to redirect CD4^+^ or CD8^+^ naïve T cells cultured in γc-cytokines into antigen-specific effector T cells. Furthermore, using this system we determined that dendritic cells are essential players in Treg-mediated suppression of T cell activation in humans.

Engineered-TCRs with known peptide-MHC specificities have been introduced into primary T cells either via co-transduction of TCRα and TCRβ chains [36] or as lentiviral vectors encoding both TCRα and β chains as a single transcript into human T cells, with improved specificity and avidity [6]. However, these approaches typically result in low expression of the engineered-TCR α/β pairs either due to transduction efficiencies or mispairing with endogenous TCR chains [6]. In this study, we sought to improve engineered-TCR expression in primary T cells by using constructed mouse-human hybrid TCRs, in which the human TCR constant regions were replaced with mouse TCR constant regions, to facilitate pairing of only ectopically introduced mouse TCR constant α/β subunits [12]. Indeed, we found that mouse-human hybrid TCR-engineered T cells were much more efficiently activated with specific peptides, compared to those expressing fully human TCR transgenes **(Figure S2)**. The CD8^+^ T cells that expressed HIV-specific TCR were also able to kill primary CD4^+^ T cells infected with HIV, suggesting these TCRs can effectively recognize endogenously processed peptides. This approach could be particularly useful in determining efficiency of larger pool of HIV-specific TCRs for optimal T cell vaccine design or for their ability to recognize latently infected CD4^+^ T cells [4], [5].

To the best of our knowledge, this is the first report demonstrating that naïve human T cells cultured in γc-cytokines, such as IL-7, which maintain their naïve state, can be transduced with specific TCRs and respond to peptide stimulation to become effector cells. Surprisingly, the naïve CD8^+^ T cells displayed significant cytotoxic potential even during the first stimulation with peptides presented by T2 cells. This cytotoxic potential of TCR-engineered CD8^+^ T cells was consistent with the induction of Granzyme B expression upon TCR-stimulation (data not shown). However, upon antigen-specific stimulation and expansion, the naïve CD8^+^ cells displayed higher magnitude of cytotoxicity at lower concentrations of peptide-stimulation compared to their naïve precursors. We propose that this novel system to induce effector/memory T cells from naïve T cells with known TCR-specificities will be a valuable tool to investigate the role of antigen density, type of APCs and affinity of the TCR to MHC-peptide complexes. This knowledge, in turn, could be important for vaccine design and cell therapy applications. Indeed, a recent report has suggested that adaptive transfer of more naïve T cells for therapeutic purposes was more sustained *in vivo* compared to effector T cells expanded *in vitro* [8].

The engineered-TCR approach represents an important advance in studying the suppressive mechanisms and activation requirements of human Tregs, since clonal expansion of these cells *in vitro* proved to be very challenging [9], [10], [25], [37]. In our experiments with TCR-engineered Tregs, we found that they were highly activated by T2 cells presenting specific peptide (gp100) as determined by induction of GARP expression. However, despite this potent TCR-stimulation, these Tregs were surprisingly inefficient at suppressing target T cell proliferation activated by the same or different peptides. However, Tregs suppressed T cell activation when DCs were used either to present the peptides to TCR-engineered Tregs or were present as bystander cells in the cultures where T2 cells were used to activate both Tregs and target cells. These findings uncover an essential role for DCs in mediating the suppressive function of Tregs. It is conceivable that DCs express additional co-stimulatory or inhibitory molecules that are not present in T2 cells. As such, we postulate that either DCs provide signals to Tregs that allow them to display their suppression or that part of the suppressive activity is relayed via DCs through their interaction with Tregs. Alternatively, DCs may facilitate Treg-suppression by attracting and clustering together the Tregs and target T cells in close proximity. We also found that, in contrast to a recent report by Plesa et al. [25], when DCs are present, Tregs are able to suppress T cell activation even when different APCs presented the different antigens. The difference could be due to differences in TCR-specificities or the use of DCs in our culture conditions. Future studies will be aimed at dissecting the mechanisms of antigen-specific Treg inhibitory activity, mediated by DCs, and molecular inputs of TCR-signaling that determine the magnitude of suppressive function.

In summary, we demonstrated that naïve T cells can be engineered with antigen-specific TCRs and that this approach can be utilized to study the role of canonical TCR engagement in their differentiation into effector subsets. Furthermore, this system revealed the important role of DCs in mediating the suppressive function of human Tregs. Therefore, this engineered-TCR system establishes a framework for future studies to investigate the mechanisms and optimal parameters of antigen-specific differentiation of human naïve T cells into memory cells, which could have therapeutic or vaccine design implications. In addition, the uses of multiple engineered-TCRs with known specificities and affinities presented by different APCs will be of importance to decode the TCR-signaling mechanisms in antigen-specific Treg suppression.

## Supporting Information

Figure S1 **Cloning strategy of SL9-TCR construct.** gp100-TCR was the mouse TCR constant αβ template#1. 1803 TCRα-2A-TCRβ was the Human TCR-SL9 variant αβ template#2. The “Step one” PCR amplification was conducted using a forward primer#1 specific for human 3′TCRvα terminal region plus mouse 5′TCRcα leader region, and a reverse primer#2 containing the sequence for mouse 3′TCRcα terminal region plus sequence coding the P2A for PCR product (PCRp1). Similar strategy was used for the amplification of PCRp2 containing human 3′TCRvβ terminal region plus mouse TCRcβ full regions by pimer#3 and #4, except in stead of using sequence coding the P2A, a sequence containing restrict enzyme Mfel was used in primer#4. The “Step two” PCR amplification was performed using a forward primer (primer#5) containing the NotI restriction site followed by 5′ human TCRvα leader sequence and a return primer, which is the PCRp1 from step one-PCR amplification to generate PCRp3. Primer#6 containing sequence complemented to P2A followed by sequence specific for human 5′TCRvβ leader region was used along with PCRp2 to amplify PCRp4. PCRp3 and PCRp4 were mixed, and the TCR-SL9 sequence was generated by “step three” PCR amplification with primer#4 and primer#5.
(TIFF)

Figure S2 **Increased cytokine production from T cells expressing mouse-human hybrid TCRs compared to fully human TCR.** CD8^+^ and CD4^+^ T cells were transduced to express engineered-human TCRs hybrid with mouse constant β region or entire human TCR (hTCR) specific for SL9 peptide. T cells were activated by SL9 through T2 cells at the concentrations indicated. IFN-γ and IL-2 from CD8^+^ and CD4^+^ T cells, respectively, were determined by CBA and FACS analysis.
(TIFF)

Figure S3 **Cytotoxicity of TCR-engineered CD8^+^ T cells based on Teff:Target ratio.** CD8_TCR-SL9_ were cultured with SL9 pulsed T2 cells at 1∶1, 1∶5, 1∶25 CD8 (Teff): T2 (Target) ratio. The % Cytotoxicity is shown. The data are representative from three different experiments from multiple donors.
(TIFF)

Figure S4 **TCR engineered-naïve T cells maintain their resting phenotype.** Freshly isolated CCR7^+^CD45RO^−^ T_N_ subset from CD8^+^ T cells were cultured in IL-7 containing medium for 7 days followed by engineered-TCRs transduction. More than 95% CD8_N TCR-SL9_ (GFP^+^) or CD8_N TCR-gp100_ (RFP^+^) cells were still CCR7^+^CD45RO^−^ at day 7 post transduction.
(TIFF)

Figure S5 **Proliferation and IL-2 secretion from Tregs_TCR-gp100_ stimulated with T2 cells**. **(A)** Tregs expressing gp100-TCR were surface stained for GARP, fixed, and then permeabilized for intracellular staining of FOXP3 and HELIOS 2 days after gp100 or MART-1 presentation by T2 cells. **(B)** Tregs_TCR-gp100_ and T_TCR-gp100_ were generated as in Figure 2, labeled with CFSE and reactivated by gp100 (10 µM) pulsed T2 cells or DCs. The proliferation was monitored at day 6 post activation and the expansion of T cells was determined at day 14 post activation. **(C)** Supernatants were collected from the same cultures after 24-hour stimulation and IL-2 levels were measured using CBA assay.
(TIFF)
