# Correction: Probing the Effector and Suppressive Functions of Human T Cell Subsets Using Antigen-Specific Engineered T Cell Receptors

**DOI:** 10.1371/annotation/cbc71d72-f1a2-45de-9d4a-cb0c8dc076b5

**Published:** 2013-10-30

**Authors:** Qi Wan, Lina Kozhaya, Keren Imberg, Frances Mercer, Shi Zhong, Michelle Krogsgaard, Derya Unutmaz

Figure S5 is missing panels B and C. Please view the correct Figure 5S here: 
