# Supplementary figures and images for: Probing the Effector and Suppressive Functions of Human T Cell Subsets Using Antigen-Specific Engineered T Cell Receptors
Source: PLoS One. 2013 Feb 20;8(2):e56302. doi: 10.1371/journal.pone.0056302 (PMC3577812; doi:10.1371/journal.pone.0056302)

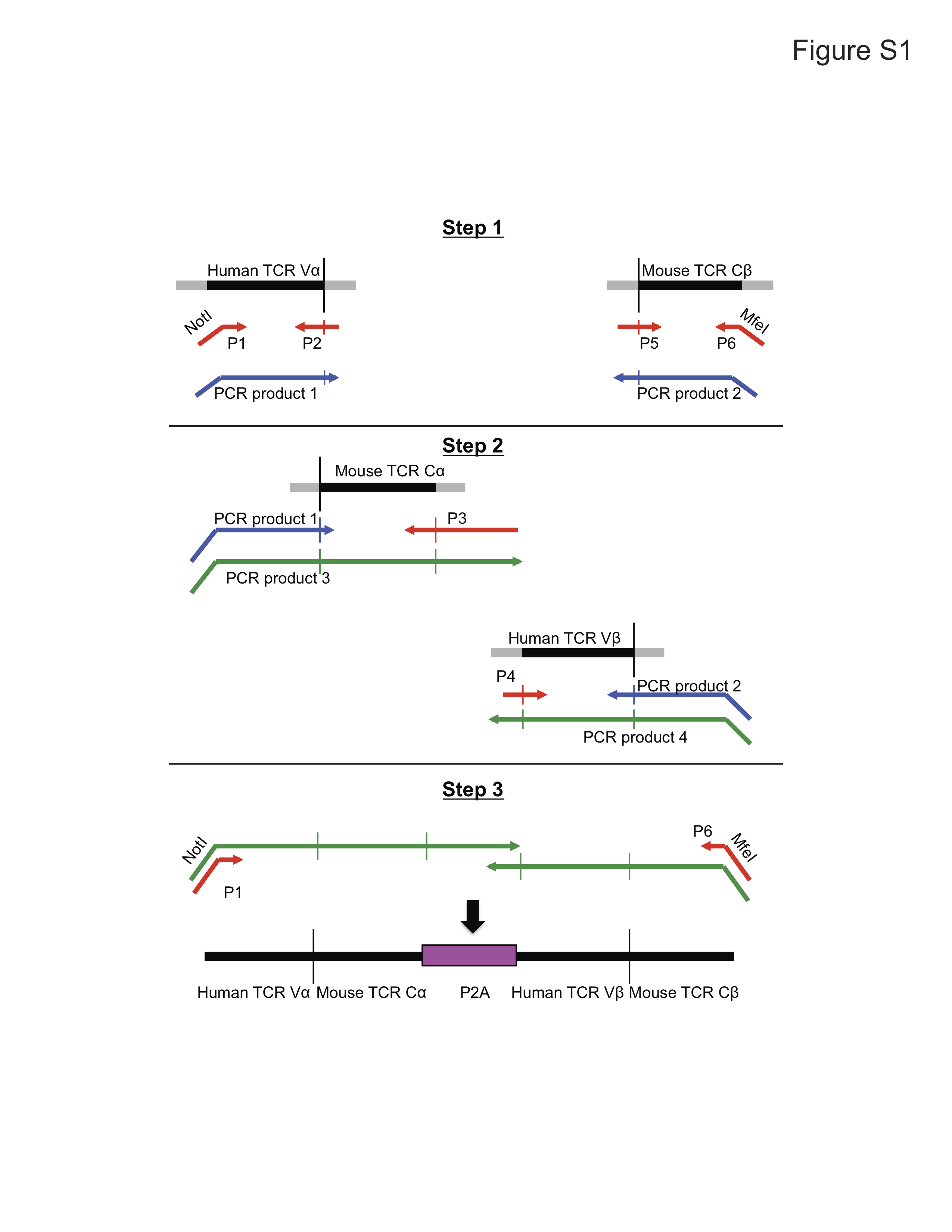

Supplement: Figure S1 — Cloning strategy of SL9-TCR construct. gp100-TCR was the mouse TCR constant αβ template#1. 1803 TCRα-2A-TCRβ was the Human TCR-SL9 variant αβ template#2. The “Step one” PCR amplification was conducted using a forward primer#1 specific for human 3′TCRvα terminal region plus mouse 5′TCRcα leader region, and a reverse primer#2 containing the sequence for mouse 3′TCRcα terminal region plus sequence coding the P2A for PCR product (PCRp1). Similar strategy was used for the amplification of PCRp2 containing human 3′TCRvβ terminal region plus mouse TCRcβ full regions by pimer#3 and #4, except in stead of using sequence coding the P2A, a sequence containing restrict enzyme Mfel was used in primer#4. The “Step two” PCR amplification was performed using a forward primer (primer#5) containing the NotI restriction site followed by 5′ human TCRvα leader sequence and a return primer, which is the PCRp1 from step one-PCR amplification to generate PCRp3. Primer#6 containing sequence complemented to P2A followed by sequence specific for human 5′TCRvβ leader region was used along with PCRp2 to amplify PCRp4. PCRp3 and PCRp4 were mixed, and the TCR-SL9 sequence was generated by “step three” PCR amplification with primer#4 and primer#5. (TIFF) [file pone.0056302.s001.tiff]

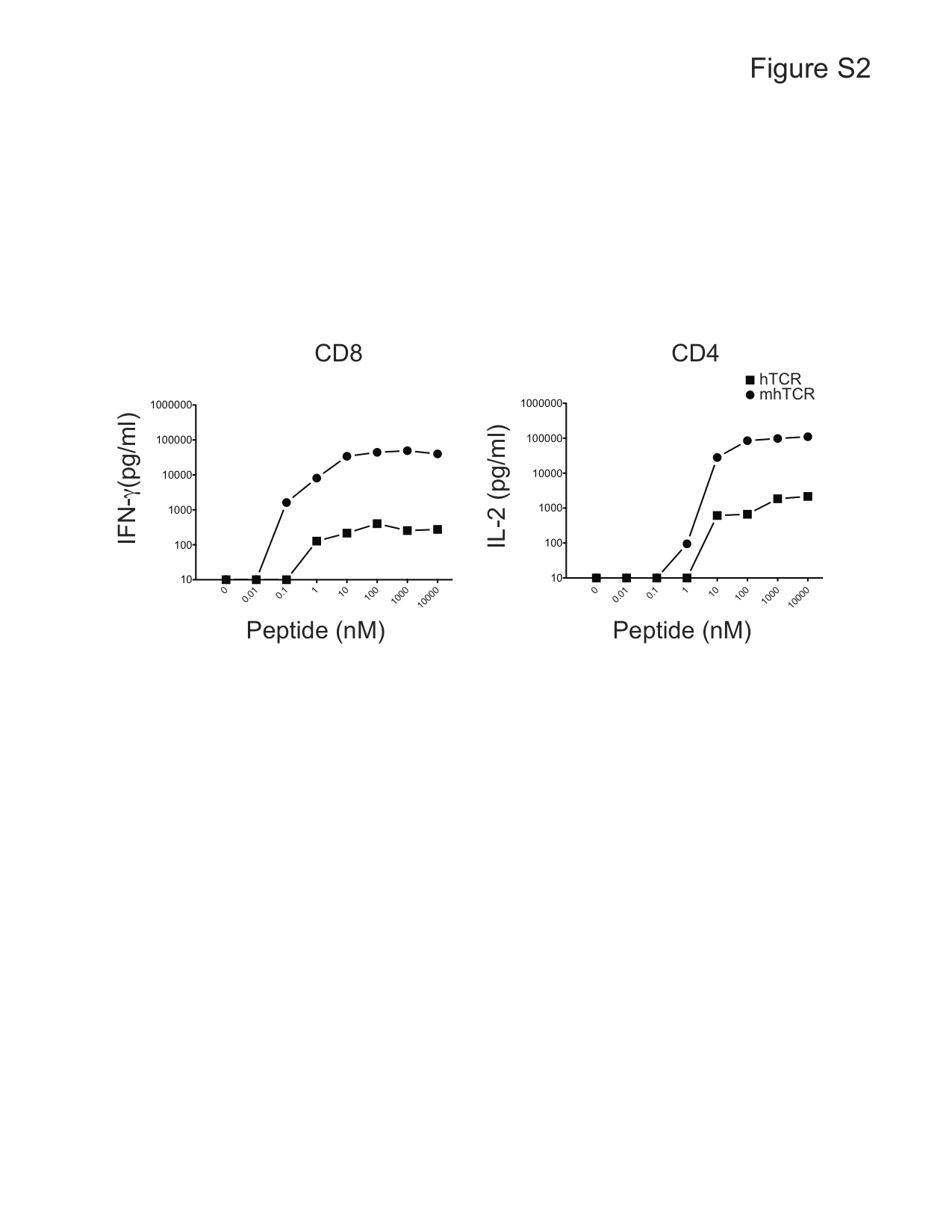

Supplement: Figure S2 — Increased cytokine production from T cells expressing mouse-human hybrid TCRs compared to fully human TCR. CD8+ and CD4+ T cells were transduced to express engineered-human TCRs hybrid with mouse constant β region or entire human TCR (hTCR) specific for SL9 peptide. T cells were activated by SL9 through T2 cells at the concentrations indicated. IFN-γ and IL-2 from CD8+ and CD4+ T cells, respectively, were determined by CBA and FACS analysis. (TIFF) [file pone.0056302.s002.tiff]

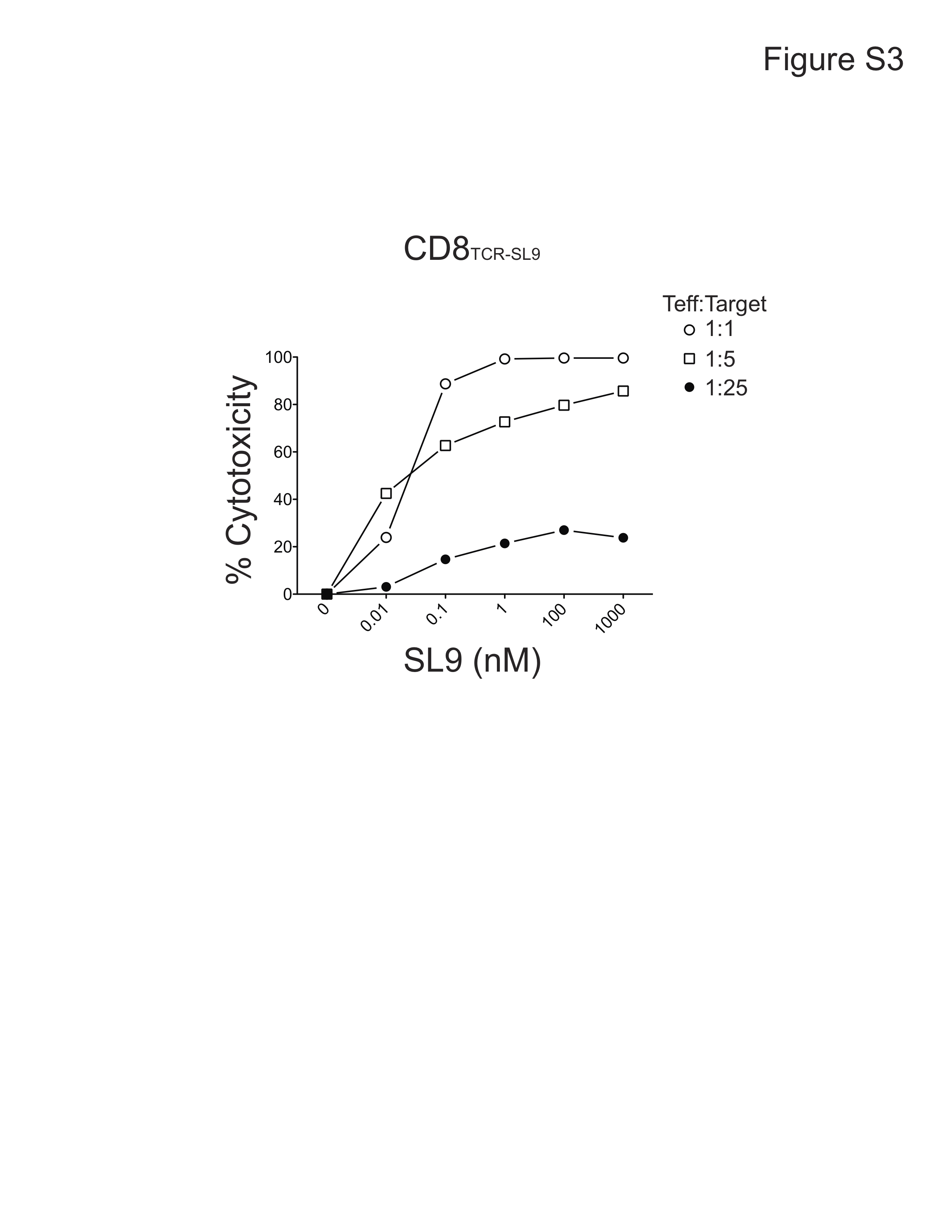

Supplement: Figure S3 — Cytotoxicity of TCR-engineered CD8+ T cells based on Teff:Target ratio. CD8TCR-SL9 were cultured with SL9 pulsed T2 cells at 1∶1, 1∶5, 1∶25 CD8 (Teff): T2 (Target) ratio. The % Cytotoxicity is shown. The data are representative from three different experiments from multiple donors. (TIFF) [file pone.0056302.s003.tiff]

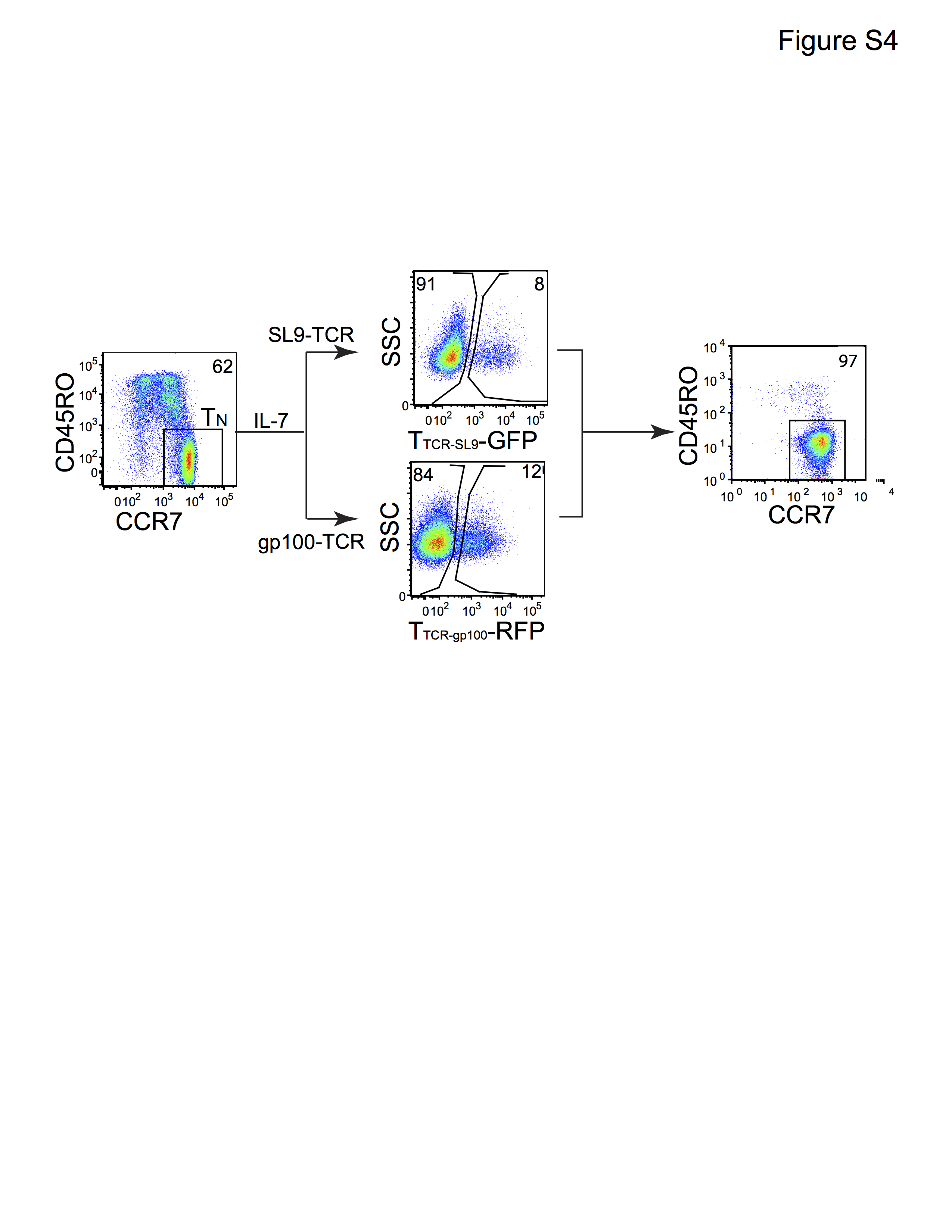

Supplement: Figure S4 — TCR engineered-naïve T cells maintain their resting phenotype. Freshly isolated CCR7+CD45RO− TN subset from CD8+ T cells were cultured in IL-7 containing medium for 7 days followed by engineered-TCRs transduction. More than 95% CD8N TCR-SL9 (GFP+) or CD8N TCR-gp100 (RFP+) cells were still CCR7+CD45RO− at day 7 post transduction. (TIFF) [file pone.0056302.s004.tiff]

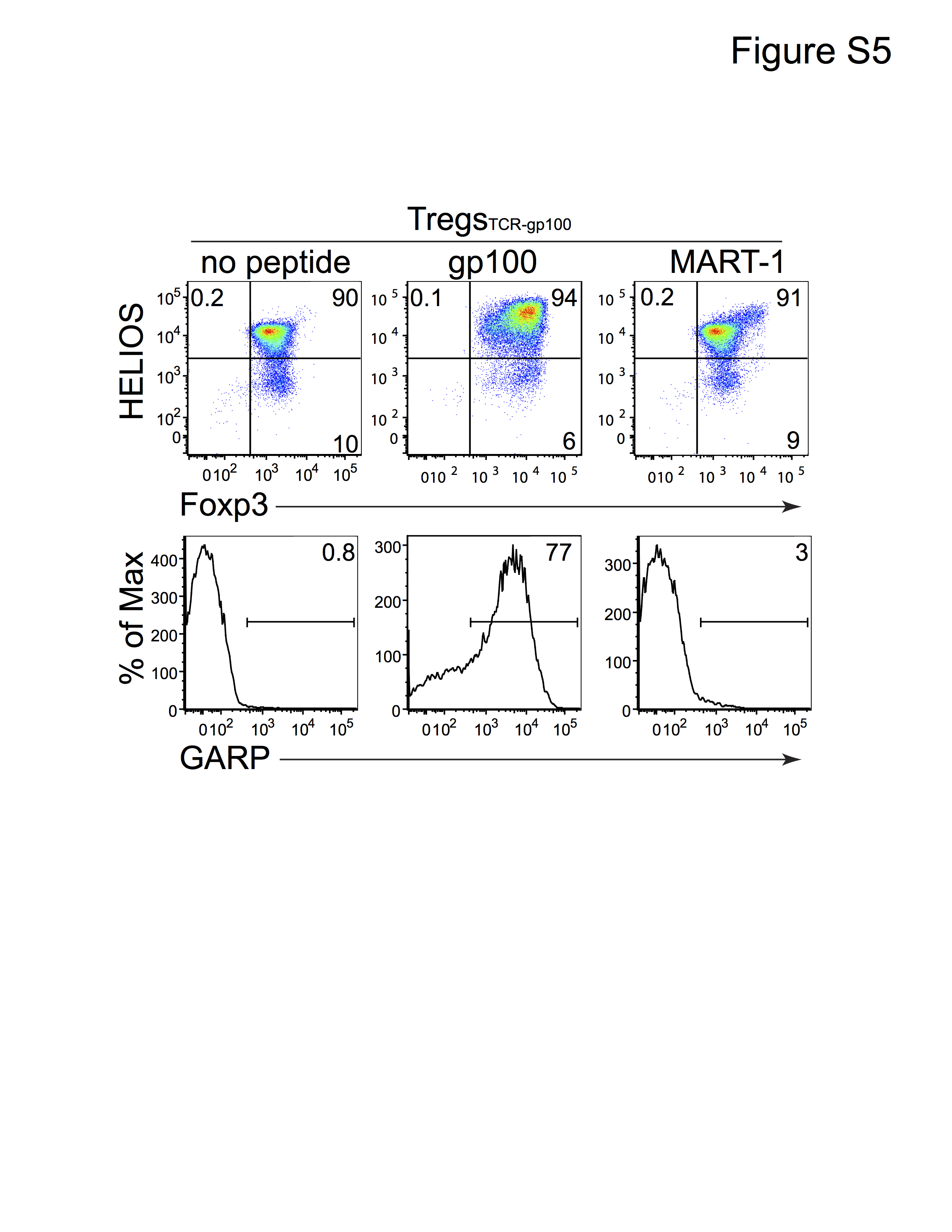

Supplement: Figure S5 — Proliferation and IL-2 secretion from TregsTCR-gp100 stimulated with T2 cells. (A) Tregs expressing gp100-TCR were surface stained for GARP, fixed, and then permeabilized for intracellular staining of FOXP3 and HELIOS 2 days after gp100 or MART-1 presentation by T2 cells. (B) TregsTCR-gp100 and TTCR-gp100 were generated as in Figure 2, labeled with CFSE and reactivated by gp100 (10 µM) pulsed T2 cells or DCs. The proliferation was monitored at day 6 post activation and the expansion of T cells was determined at day 14 post activation. (C) Supernatants were collected from the same cultures after 24-hour stimulation and IL-2 levels were measured using CBA assay. (TIFF) [file pone.0056302.s005.tiff]
