# Supplementary figures and images for: Correction: Probing the Effector and Suppressive Functions of Human T Cell Subsets Using Antigen-Specific Engineered T Cell Receptors
Source: PLoS One. 2013 Oct 30;8(10):10.1371/annotation/cbc71d72-f1a2-45de-9d4a-cb0c8dc076b5. doi: 10.1371/annotation/cbc71d72-f1a2-45de-9d4a-cb0c8dc076b5 (PMC3815353; doi:10.1371/annotation/cbc71d72-f1a2-45de-9d4a-cb0c8dc076b5)

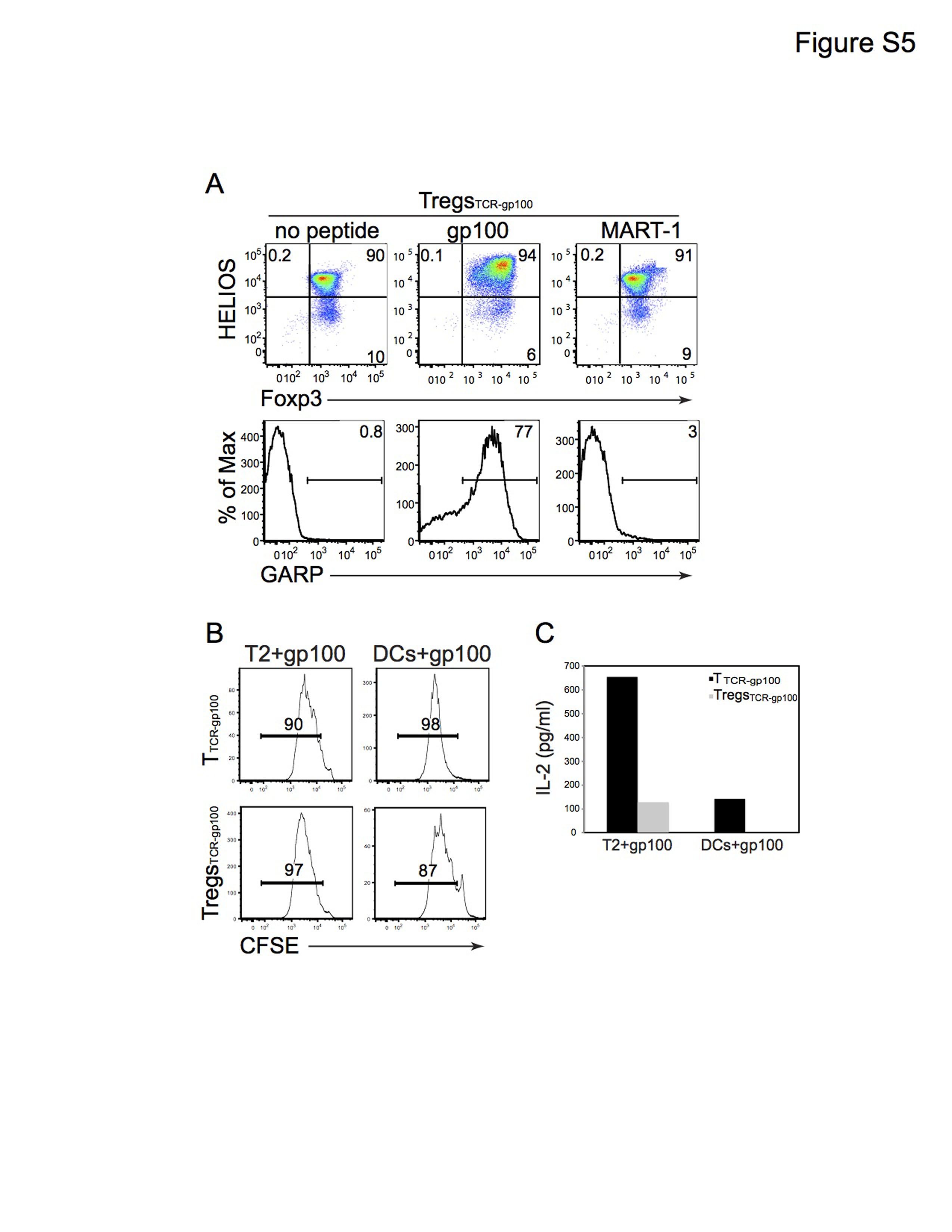

Supplement: Supplementary file 1 [file pone.cbc71d72-f1a2-45de-9d4a-cb0c8dc076b5.s001.jpg]
